# Hyperthermia induced HIF-1a expression of lung cancer through AKT and ERK signaling pathways

**DOI:** 10.1186/s13046-016-0399-7

**Published:** 2016-07-26

**Authors:** Jun Wan, Wei Wu

**Affiliations:** 1 Department of Thoracic Surgery, The First Affiliated Hospital of Anhui Medical University, NO.218, Jixi Road, Hefei, 230022 Anhui People’s Republic of China; 2 Department of Hematology, The First Affiliated Hospital of Anhui Medical University, Hefei, 230022 China

**Keywords:** Angiogenesis potential, Cd34, Extracellular regulated protein kinases (erk), Hyperthermia, Hypoxia-inducible factor-1 alpha (hif-1a), Non small cell lung cancer (nsclc), Protein kinase b (akt), Small cell lung cancer (sclc), Signaling pathway protein, Tumor proliferation

## Abstract

**Background:**

Hyperthermia is a promising treatment for human lung cancer, but recurrence of the primary lesion is common, as the residual tumor becomes adapted to heat treatment and growth is induced by hypoxia-triggered HIF-1a expression. Here, we explored the effects of hyperthermia on HIF-1a expression, proliferation, and lung cancer angiogenesis.

**Methods:**

Human NSCLC NCI-H1650 and SCLC NCI-H446 cell lines were used to examine cell viability, apoptosis, and HIF-1a expression level under a gradient of thermal conditions (37, 42 and 47 °C for 40 min). The 47 °C heat-adapted NCI-H1650 and NCI-H446 sublines (also called NCI-H1650-b and NCI-H446-b cells) had enhanced viability and HIF-1a expression levels compared to the parental and 42 °C heat-adapted cells and were thus used for subsequent research. Concentration gradients of wortmannin and PD98095 were used to inhibit AKT and ERK expression, respectively in the NSCLC NCI-H1650-b and SCLC NCI-H446-b cell lines, and cell growth curves were drawn. Western blots were used to detect the expression of HIF-1a, extracellular signal-regulated kinase (ERK), protein kinase B (AKT), phospho-ERK, and phospho-AKT. We established a subcutaneous transplantation tumor model with wortmannin and PD98095 intervention. Immunohistochemistry was used to detect the expression of HIF-1a and the vascular specific marker CD34, and tumor growth curves were drawn.

**Results:**

Following hyperthermia treatment, HIF-1a expression in 47 °C heat-adapted NSCLC and SCLC cell lines was regulated by the AKT pathway. However, HIF-1a expression was also regulated by the ERK pathway in NSCLCs, while SCLCs did not exhibit changes in ERK. These biological behaviors are governed by signaling pathway protein phosphorylation. Furthermore, inhibiting the AKT pathway can suppress the proliferation and angiogenesis potential of both 47 °C heat-adapted NSCLCs and SCLCs, but inhibiting the ERK pathway only affects SCLCs.

**Conclusion:**

Our study suggests that following hyperthermia, the proliferation and angiogenesis potential of residual NSCLCs and SCLCs is induced by HIF-1a. However, HIF-1a expression in NSCLCs is regulated by both the AKT and ERK signaling pathway, but HIF-1a expression in SCLCs is regulated only by the AKT signaling pathway. This study sheds light on the molecular regulatory mechanisms of lung cancer recurrence following hyperthermia treatment.

## Background

In recent years, hyperthermia has been recognized as a clinical treatment for certain cancers (e.g. lung cancer), which can be induced by therapeutic techniques, such as radiofrequency ablation (RFA). However, one of the major problems with hyperthermia is that it is difficult to achieve complete tumor destruction using this technique, and recurrence is common, because the residual tumor is now adapted to heat treatment and growth is induced [1]. Accumulating evidence indicates that both hypoxia and hypoxia-driven angiogenesis are the consequence of hyperthermia, and both of these factors play important roles in tumor growth [2]. Under conditions of hypoxia, a signaling pathway involving a crucial oxygen response regulator, defined hypoxia-inducible factor (HIF), is turned on. HIF protein, especially HIF-1α and HIF-2α, are correlation with tumor development, metastasis and promote epithelial-mesenchymal transition [3]. In contrast with HIF-2α, which is expressed in certain cell types of vertebrate species, the expression of HIF-1α is observed in most metazoan species and involved in the regulation of epithelial-mesenchymal transition of tumor [4]. As a key transcriptional regulator, HIF-1α plays a central role in the adaptation of tumor cells to hypoxia and helps to regulate the expression of muitiple cytokines, such as vascular endothelial growth factor-A (VEGF-A), and promotes the proliferation [5] and angiogenesis potential [6] of small cell lung cancers (SCLCs). Earlier research also showed that HIF-1a is involved in apoptosis and the proliferation of non-small cell lung cancers (NSCLCs) [7, 8]. In our previous study, we found that local recurrences of SCLC following RFA treatment were driven by HIF-1a, while the thermal effects of RFA can promote the growth of residual NSCLCs by up-regulating HIF-1a expression [9].

The expression and activity of HIF-1a is not only induced in response to limited oxygen availability, but it is also modulated through related signaling pathways [10]. Some studies have demonstrated that the expression of HIF-1a is regulated by major signaling pathways, including the extracellular signal-regulated kinase (ERK) pathway [11] and the protein kinase B (AKT) pathway [12]. Intensive studies of the ERK and AKT pathways have revealed these signaling pathways play their most important roles in the molecular signaling network that governs growth, proliferation, differentiation and survival in many, if not all, cell types [13, 14]. Therefore, to uncover the molecular mechanisms of lung cancer recurrence following hyperthermia treatment, the present study explores the effects of hyperthermia on HIF-1a expression and cell proliferation and angiogenesis. We then went on to investigate the regulation and function of the Akt and ERK signaling pathways.

## Methods

### Chemicals and antibodies

Specific inhibitor wortmannin, PD98059, and Recombinant Human Heregulin were purchased from Sigma, St. Louis, MO, USA. TRIzol reagent was purchased from Invitrogen, Carlsbad, CA. RIPA lysis buffer was purchased from the Beyotime Institute of Biotechnology, China. The following primary antibodies: anti-CD34 (1:40 dilution), anti-HIF-1a (1:500 dilution), anti-ERK1/2 (1:1000 dilution), and anti-AKT (1:1000 dilution) were purchased from Wuhan Boster Biological Engineering Technology Limited Company. Anti-phospho-AKT (1:1000 dilution) was purchased from Cell Signaling Technology, Beverly, MA, USA. and anti-phospho-ERK1/2 (1:800 dilution) was purchased from Santa Cruz, CA, USA.

### Cell culture, heat treatment of cells, and establishment of sublines

Human NSCLC NCI-H1650 cells and SCLC NCI-H446 cells were maintained in RPMI-1640 medium (Sigma-Aldrich Co., St. Louis, MO, USA) supplemented with 10 % fetal bovine serum (FBS), 100 units/mL penicillin, and 100 μg/ml kanamycin at 37 °C in a humidified atmosphere containing 5 % CO_2_ and 20 % O_2_. The medium was routinely changed 2-3 days after seeding. Cells were detached with trypsin/EDTA (GibcoBRL, Paisley, UK) and were resuspended in a 1:1 solution of serum-free RPMI-1640 medium to a final concentration of approximately 5× 10^5^ cells/10 μl.

During the exponential phase, cells were exposed to hyperthermic stress in cell culture plates for 24, 48, or 72 h. The plates were sealed with parafilm and submerged in a water bath at the desired temperature for 10 min. The three desired temperatures were 37, 42, and 47 °C. The cells cultured at 42 and 47 °C generated 2 cell sublines each, named NCI-H1650-a and NCI-H1650-b; NCI-H446-a and NCI-H446-b respectively. After the hyperthermic treatment, fresh culture medium was added to each well, and the surviving cells were maintained at 37 °C with an atmosphere containing 5 % CO_2_ and 1 % O_2_ for 4 h. After determining cell viability and HIF-1a expression levels, we chose one subline for the following experiment.

### TUNEL staining for apoptosis assay

Cells were grown on coverslips, treated with 5 μM BIX for 48 h and stained with Click-iT plus TUNEL assay kit (Invitrogen) according to previous study [15]. Then, cells were washed with PBS, fixed in 4 % paraformaldehyde (PFA) for 20 min, and permeabilized with 0.25 % Triton X-100 for 15 min. The cells were incubated with TdT reaction buffer for 10 min, then TdT reaction mixture for 1 h, and then incubated with TUNEL reaction cocktail for 30 min. Incubation with 0.05 % Diaminobenzidine tetrahydrochloride (DAB) solution for 8 min was used for counterstaining. Cells were examined using a Nikon microscope with Image-Pro Plus 6.x software (Diagnostic Instruments, USA) for image analysis.

### MTT assay for lung cancer cells viability

Cells were cultured at a concentration of 1 × 10^4^ cells/well in 48-well plates. The 3-(4,5-dimethylthiazol-2-yl)-2,5-diphenyl tetrazolium bromide (MTT) solution was added to each well at a final concentration of 0.5 mg/ml and incubated for 4 h. At the end of the incubation, formazan crystals, resulting from MTT reduction, were dissolved by addition of 150 ml DMSO per well. The optical density was read at 570 nm, and the average values were determined from replicate wells.

### Growth of xenografts in nude mice

Male congenital athymic BALB/c nude mice were obtained from the Experimental Animal Center of the Shang Hai Jiao Tong University School of Medicine. They were maintained under pathogen-free conditions in accordance with established institutional guidance and approved protocols. All experiments were carried out using 6-8-week-old mice weighting 16-22 g. Sublines of NCI-H1650 and NCI-H446 cells were cultured in vitro (1 × 10^7^), and a final concentration of approximately 5 × 10^5^ cells/10 μl were suspended in PBS and subcutaneously injected into the flank area of mice. After tumors reached 3-5 mm in diameter, mice were injected with either vehicle (10 % DMSO/PBS), 4 mg/kg, or 5 mg/kg twice weekly. The tumor size was measured with calipers every 3 days, and tumor volume was calculated according to the formula: volume = width^2^ × length × 0.5. Tumors were removed and weighed 30 days after inoculation. All surgical procedures were performed under isoflurane inhalation anesthesia. Buprenorfine was injected intramuscularly prior to surgery for perioperative analgesia.

### Immunohistochemistry detection for HIF-1a and vascular specific markers

All tumor tissue sections were cut into 4 μM sections, deparaffinized, and endogenous peroxidases were inhibited with 0.3 % hydrogen peroxide in methanol for 30 min. Antigen retrieval was achieved using 0.05 % protease XIV at 37 °C for 5 min. Sections were then incubated with a mouse anti-human HIF-1a or CD34 primary antibody overnight at 4^o^C. Next, the slides were incubated with biotin-conjugated rabbit anti-mouse secondary antibody at room temperature for 45 min. The sections were subsequently incubated with a streptavidin-biotin-peroxidase complex (Vectastain ABC kit, Vector Laboratories, Burlingame, CA, USA) at room temperature for 45 min. The reaction was visualized using chromogen diaminobenzidine (DAB) for 10 s. Finally, the slides were counterstained with hematoxylin and mounted. The slides were examined with a Nikon Eclipse Ti microscope under a 40X objective.

### Western-blot analysis of HIF-1a and signaling protein

Cells and tissues were harvested and analyzed for the expression of HIF-1a, ERK, p-ERK, AKT, and p-AKT. Briefly, total protein was extracted by disrupting cells in RIPA lysis buffer and separating on a polyacrylamide gel and transferring to PVDF membrane. The membranes were then blocked at room temperature for 1 h with 5 % non-fat milk in Tris buffered saline containing Tween 20 (TBST). The membrane was incubated with anti-HIF-1a, anti-ERK1/2, anti-AKT, anti-phospho-AKT, and anti-phospho-ERK1/2 primary antibodies at 37^o^C for 2 h, and then with peroxidase-conjugated IgG at room temperature for 1 h. The membranes were subsequently incubated with goat anti-rabbit peroxidase-conjugated secondary antibodies, and immunoreactivity was detected by using an enhanced chemiluminescence kit, and captured on X-ray film. β-actin was used as an internal control.

### Statistical analysis

The SPSS 13.0 software (SPSS, USA) was applied to complete data processing. An independent-samples t-test was used to evaluate the differences in optical density (OD) values or tumor cell numbers between groups with various treatments. All data are represented as the mean ± SD for three independent experiments. Results were considered statistically significant when the p-value was less than 0.05.

## Results

### In vitro heat treatment can generate NCI-H1650 cell sublines with increased viability

**Fig. 1:**
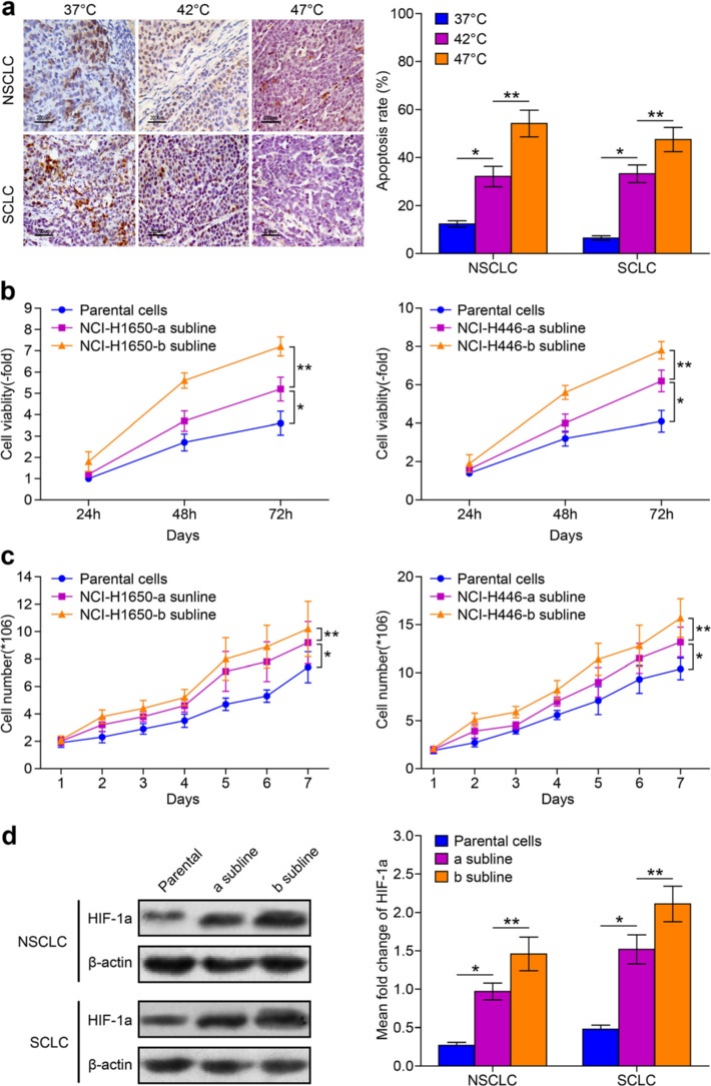
The viability of NCI-H1650 cells and NCI-H446 cells and their sublines adapted to hyperthermia treatment. **a** NCI-H1650 and NCI-H446 cells are cultured under 37, 42, and 47 °C. Apoptosis rate is measured using a Tunnel staining assay. The brown stained cells are apoptocic cells, and semi-quantitative analysis of apoptosis rates are measured. (^*^ *p* < 0.05 37 °C vs 42 °C heat treatment group, ^**^ *p* < 0.05 47 °C vs 42 °C heat treatment group) (**b**) 2 sublines were established following incubation at 42 and 47 °C. The 24, 48, and 72 h viability was evaluated by MTT assay. Parental cells: cells cultured under 37 °C; NCI-H1650-a and NCI-H446-a subline: cells adapted to 42 °C heat treatment; NCI-H1650-b and NCI-H446-b subline: cells adapted to 47 °C heat treatment (^*^ *p* < 0.05 NCI-H1650-a or NCI-H446-a subline vs parental cells, ^**^ *p* < 0.05 NCI-H1650-a subline vs NCI-H1650-b subline, NCI-H446-a subline vs NCI-H446-b subline) (**c**) Growth curve of parental cells and the cells subline a and b are drawn. Data are the representative results of three independent experiments (^*^ *p* < 0.05 from day 2-7 parental cells vs cells subline-a, ^**^ *p* < 0.05 from day 2-7 cells subline-a vs cells subline-b). **d** HIF-1a expression of parental cells, the cell subline a and b are detected by Western-blot and semi-quantitative analysis. (^*^ *p* < 0.05 parental cells vs cells subline-a, ^**^ *p* < 0.05 cells subline-a vs cells subline- b)

To detect the potential effect of hyperthermia on the proliferative activity of lung cancer cells, first we monitored cell viability at 24, 48, and 72 h after incubation at 37, 42, or 47 °C for 10 min. We found that the apoptosis rate of NCI-H1650 and NCI-H446 cells after incubation at 47 °C was significantly higher compared to cells incubated at 37 °C or 42 °C (Fig. 1a). In addition, the apoptosis rate of cells incubated at 42 °C was higher than at 37 °C. The cells cultured at conventional culture temperature (37 °C) are called parental cells, and the cells cultured and adapted to 42 and 47 °C were named the NCI-H1650-a and NCI-H446-a and NCI-H1650-b and NCI-H446-b sublines respectively. The cellular viability of parental cells and sublines all reach their highest viability at 72 h. However, the cell viability of sublines adapted to 47 °C are higher than the sublines adapted to 42 °C or the parental cells. The viability of sublines adapted to 42 °C are higher than parental cells (Fig. 1b). From the growth curve, we can see that the proliferation activity of sublines adapted to 47 °C are higher than the sublines adapted to 42 °C or the parental cells (Fig. 1c). Furthermore, the HIF-1a expression level of sublines adapted to 47 °C is also higher than the subline adapted to 42 °C or the parental cells (Fig. 1d). Therefore, we conclude that thermal treatment can partially kill lung cells, while generating sublines with increased viability with higher HIF-1a expression levels. Therefore, we used the NCI-H1650 subline adapted to 47 °C for our subsequent experiments.

### Specific inhibitor wortmannin inhibited HIF-1a expression, and the proliferation, and angiogenesis potential of NSCLCs and SCLCs following 47 °C heat treatment

**Fig. 2:**
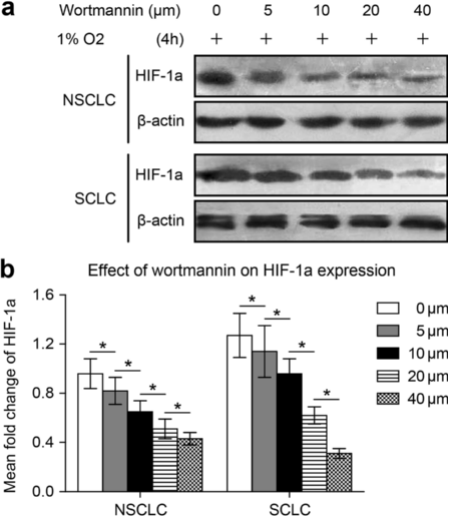
Wortmannin inhibits HIF-1a expression in NSCLC and SCLC cells following 47 °C heat treatment. NCI-H1650-b and NCI-H446-b sublines are cultured to 80-90 % confluence and exposed to hypoxia. **a** NCI-H1650-b and NCI-H446-b sublines are pretreated with different concentrations of wortmannin exposed to 1 % oxygen. HIF-1a protein expression level is measured by Western blot. **b** The result of semi-quantitative analysis show that with increasing concentrations of wortmannin, HIF-1a expression in the NCI-H1650-b and NCI-H446-b cell sublines decreased gradually (^*^ *p* < 0.05 between different groups)

**Fig. 3:**
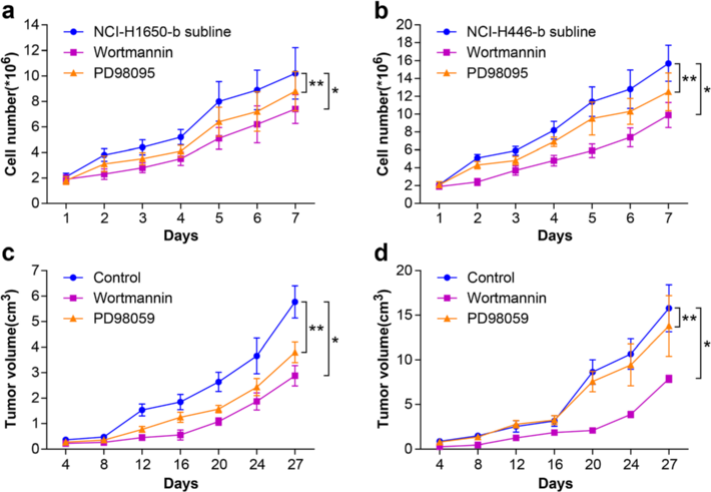
Effect of Wortmannin or PD98095 on the proliferation of NSCLC and SCLC following heat treatment. In vitro, the NCI-H1650-b and NCI-H446-b sublines (in logarithmic phase) are treated with wortmannin or PD98095 (40 μM). We compared the growth curve of the cells in control group, wortmannin group, and PD98095 group. **a** When the NCI-H1650-b subline was treated with wortmannin or PD98095, the growth curve shifted to the right and the growth rate was slowed (^*^ *p* < 0.05 wortmannin group vs control group, ^**^ *p* < 0.05 PD98095 group vs control group). **b** When the NCI-H446-b subline was treated with wortmannin, the growth curve shifted to right and the growth rate was significantly slowed (^*^ *p* < 0.05 wortmannin group vs control group), but after treatment with PD98095, the growth curve changed very little (^**^ *p* > 0.05 PD98095 group vs control group). **c** The growth curve of subcutaneous tumors formed by the NCI-H1650-b subline show that after treatment with wortmannin or PD98095, the curve moved right (^*^ *p* < 0.05 wortmannin group vs control group, ^**^ *p* < 0.05 PD98095 group vs control group). **d** The growth curve of SCLC transplantation tumors shows that treatment with wortmannin but not PD98095 causes the rightward movement of growth curve (^*^ *p* < 0.05 wortmannin group vs control group, ^**^ *p* > 0.05 PD98095 group vs control group)

**Fig. 4:**
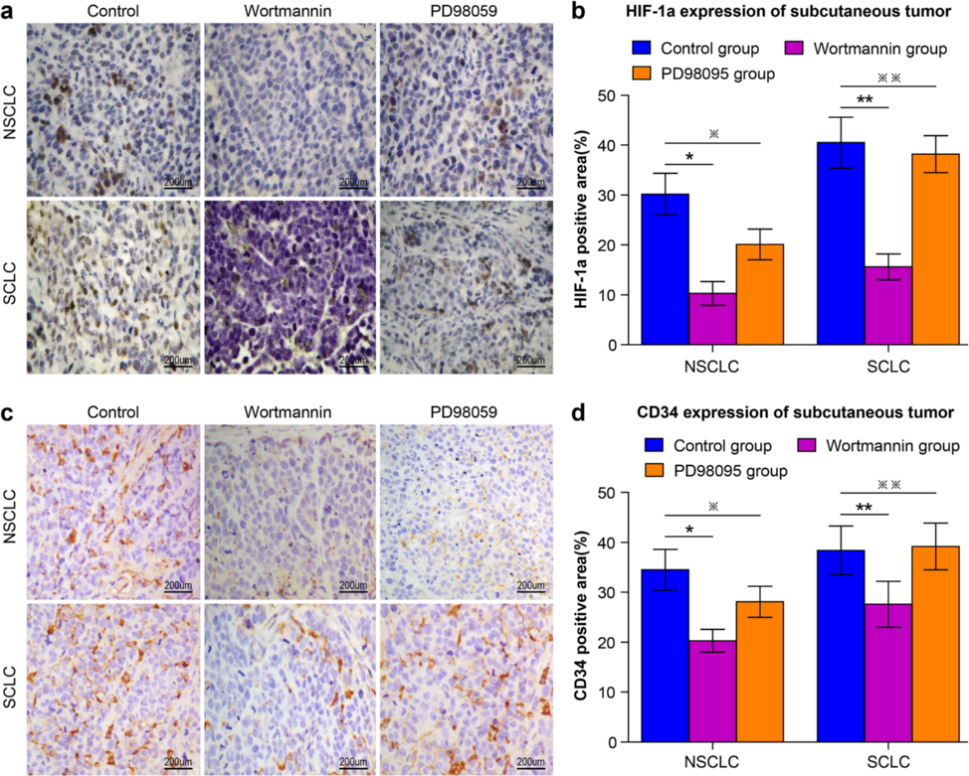
The effect of wortmannin and PD98095 on HIF-1a expression and angiogenesis potential in NSCLC and SCLC subcutaneous tumors following heat treatment. NCI-H1650-b and NCI-H446-b sublines are subcutaneously injected into nude mice to form transplantation tumors. **a** Analysis of immunohistochemistry indicates HIF-1a expression in NSCLC and SCLC subcutaneous tumor before and after treatment with wortmannin or PD98095. **b** According to the results of semi-quantitative analysis, HIF-1a expression in NSCLCs following heat treatment is inhibited after treatment with wortmannin or PD98095. HIF-1a expression in SCLCs is inhibited by wortmannin but does not change significantly with PD98095 treatment (HIF-1a in control group vs wortmannin group: NCI-H1650-b ^*^ *p* < 0.05, NCI-H446-b ^**^ *p* < 0.05, HIF-1a in control group vs PD98095 group: NCI-H1650-b ^※^ *p* < 0.05, NCI-H446-b ^※※^ *p* > 0.05) (**c**) Analysis of immunohistochemistry indicates changes in CD34 expression in NSCLC and SCLC subcutaneous tumors before and after treatment with wortmannin or PD98095. **d** The results of semi-quantitative analysis show that CD34 expression in NSCLCs following heat treatment is inhibited after treatment with wortmannin or PD98095. CD34 expression in SCLCs is inhibited by wortmannin, but changes little after treatment with PD98095 (CD34 in control group vs wortmannin group: NCI-H1650-b ^*^ *p* < 0.05, NCI-H446-b ^**^ *p* < 0.05, CD34 in control group vs PD98095 group: NCI-H1650-b ^※^ *p* < 0.05, NCI-H446-b ^※※^ *p* > 0.05)

We investigated the effect of Akt signaling on HIF-1a expression in both NSCLC NCI-H1650 cells and SCLC NCI-H446 cells adapted to 47 °C. Because HIF-1a expression in the NCI-H1650-b and NCI-H446-b sublines was elevated, we cultured the cells under hypoxic conditions (1 % oxygen concentration). Wortmannin, a specific inhibitor of the Akt signaling pathway, reduced HIF-1a protein expression in a dose-dependent manner in both the NCI-H1650-b and NCI-H446-b cell sublines (Fig. 2a, b). From the cell growth curve, we found that the proliferation of the NCI-H1650-b and NCI-H446-b sublines was inhibited by wortmannin (Fig. 3a, b). To further evaluate the effect of AKT signaling on HIF-1a expression and angiogenesis potential, we next examined the effects of wortmannin on NCI-H1650-b and NCI-H446-b subline xenografts. NCI-H1650-b and NCI-H446-b subline cells were injected into the flanks of athymic BALB/c nude mice to facilitate development of a xenograft. One week following injection of the tumor cells, wortmannin mixed with 5 % sodium bicarbonate was intraperitoneally delivered daily for three weeks. We found that the growth of the tumor xenografts was significantly inhibited by wortmannin (Fig. 3c, d), while immunohistochemical staining showed HIF-1a and CD34 expression was significantly decreased (Fig. 4a-d).

### Specific inhibitor PD98095 inhibited HIF-1a expression, proliferation, and angiogenesis potential in NSCLCs but not SCLCs following 47 °C heat treatment

**Fig. 5:**
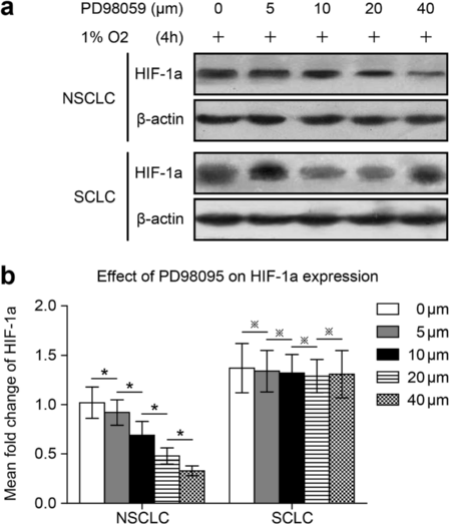
PD98095 inhibits HIF-1a expression in NSCLC cells but not SCLC cells following heat treatment. After cultured to 80-90 % confluence, NCI-H1650-b and NCI-H446-b cell sublines are exposed to hypoxia and harvested to determine HIF-1a expression levels. **a** NCI-H1650-b and NCI-H446-b cell sublines are also treated by different concentrations of PD98095 exposed to 1 % oxygen. HIF-1a protein expression level is measured by Western blot. **b** The result of semi-quantitative analysis shows that with increasing concentrations of PD98095, HIF-1a expression in NCI-H1650-b sublines decreases gradually (^*^ *p* < 0.05 between different groups), and the greatest degree of inhibition occurred at a concentration of 40 μM. However, the HIF-1a expression level of the NCI-H446-b subline was not affected by the concentration changes in PD98095 (^※^ *p* > 0.05 between different groups)

To determine if ERK signaling affected HIF-1a expression in NSCLC NCI-H1650 and SCLC NCI-H446 cells adapted to 47 °C, we used the ERK specific inhibitor, PD98095. PD98095 inhibited hypoxia-induced HIF-1a protein expression in a dose-dependent manner in NCI-H1650-b cells (Fig. 5a, b), while inhibiting cell proliferation (Fig. 3a). On the contrary, HIF-1a expression was not inhibited by PD98095 in NCI-H1650-b cells (Fig. 5a, b); proliferation of these cells was also unaffected (Fig. 3b). After the establishment of NCI-H1650-b and NCI-H446-b subline xenografts, we injected PD98095 mixed with 5 % sodium bicarbonate daily for three weeks. We found that the growth of the NSCLC tumor xenografts was inhibited by PD98095 (Fig. 3c), while immunohistochemical staining showed that HIF-1a and CD34 expression was significantly decreased (Fig. 4a-d). However, PD98095 had no obvious effect on the growth of the SCLC tumor xenografts (Fig. 3d), and HIF-1a and CD34 expression in this tumor tissue showed only marginal changes (Fig. 4a-d).

### HIF-1a expression in the NSCLC cell subline adapted to 47 °C was induced by both AKT and ERK signaling

**Fig. 6:**
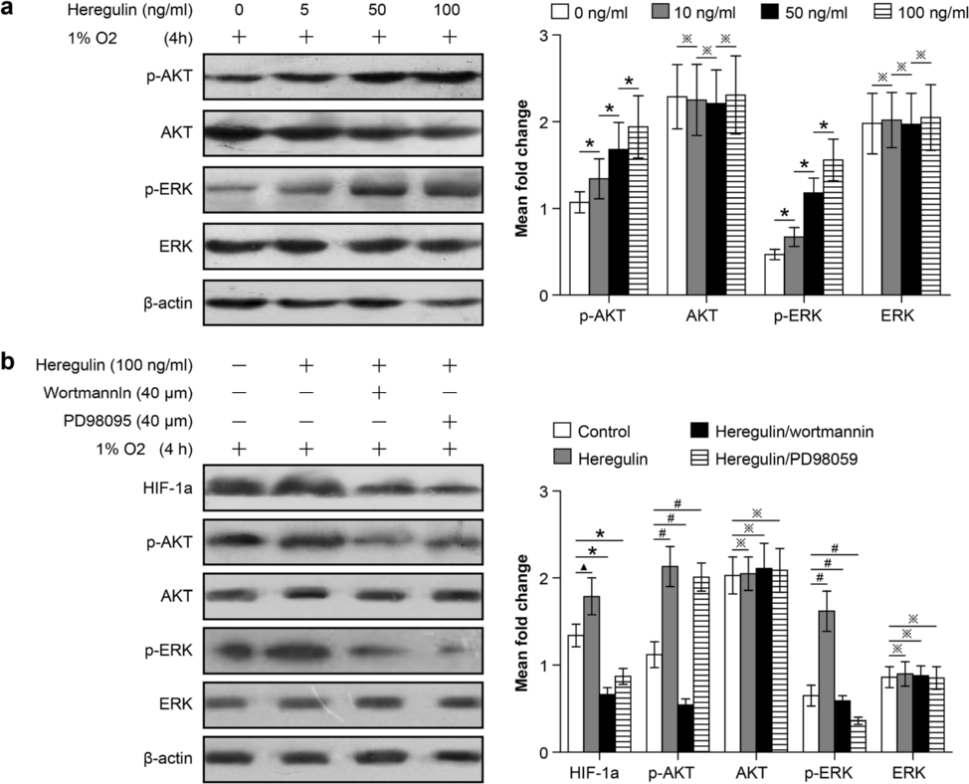
HIF-1a expression in NSCLCs following heat treatment is regulated through both the AKT and ERK signaling pathways. NCI-H1650-b sublines are seeded and pretreated with different concentrations of heregulin and then exposed to 1 % oxygen. Expression levels of AKT, ERK, phosphorylated AKT, and phosphorylated ERK were determined by western blot analysis. **a** After treatment with heregulin, AKT and ERK total protein expression levels showed no obvious change (^*^ *p* > 0.05 between different groups), but p-AKT and p-ERK were significantly up-regulated (^※^ *p* < 0.05 between different groups). **b** NCI-H1650 –b cells are seeded and pretreated with wortmannin and PD98059, then treated with heregulin. Cells are harvested and protein is detected by western blotting. When treated with heregulin, HIF-1a expression is significantly up-regulated (^▲^ *p* < 0.05 heregulin group vs control group), but is inhibited after co-treatment with wortmannin or PD98059 (^*^ *p* < 0.05 heregulin group vs heregulin + wortmannin group or heregulin + PD98059 group). Total AKT and ERK protein showed no significant changes, whether treated singly or co-treated with wortmannin or PD98059, according to the analysis of optical density (^※^ *p* > 0.05 heregulin group vs control group, heregulin + wortmannin group or heregulin + PD98059 group). However, p-AKT and p-ERK expression was up-regulated when cells were treated with heregulin, but was inhibited after co-treatement with wortmannin or PD98059 (^#^ *p* < 0.05 heregulin group vs control group, heregulin + wortmannin group or heregulin + PD98059 group)

We next studied the signaling pathways regulating the proliferation and angiogenesis potential of NSCLC cells adapted to 47 °C. Previous reports confirmed that AKT and ERK signaling pathways are involved in regulating the important biological characteristics of tumors derived from multiple tissue sources. To characterize the roles of AKT and ERK in regulating HIF-1a expression, wortmannin and PD98059 were used again. NCI-H1650-b cells were cultured under hypoxic conditions, following treatment by heregulin, which induces HIF-1a expression [16]. We found that AKT and ERK total expression levels did not change much with treatment, but the abundance of their phosphorylated forms, p-AKT and p-ERK, increased in a dose dependent manner (Fig. 6a). These results indicate that the phosphorylated forms of AKT and ERK play a major role in the regulation of HIF-1a expression. To confirm this finding, we pre-treated NCI-H1650-b cells with the heregulin and then treated with wortmannin or PD98095 and incubated under hypoxic conditions for 6 h. Our study showed that wortmannin abolishes heregulin-induced HIF-1a expression, and PD98059 also significantly attenuates HIF-1a expression (Fig. 6b). Taken together, these results indicate that the inactivation of the AKT and ERK signaling pathways is important for the inhibition of HIF-1a expression in NSCLCs following hyperthermic treatment.

### HIF-1a expression of the SCLC cell subline adapted to 47 °C is induced by the AKT, but not the ERK signaling pathway

**Fig. 7:**
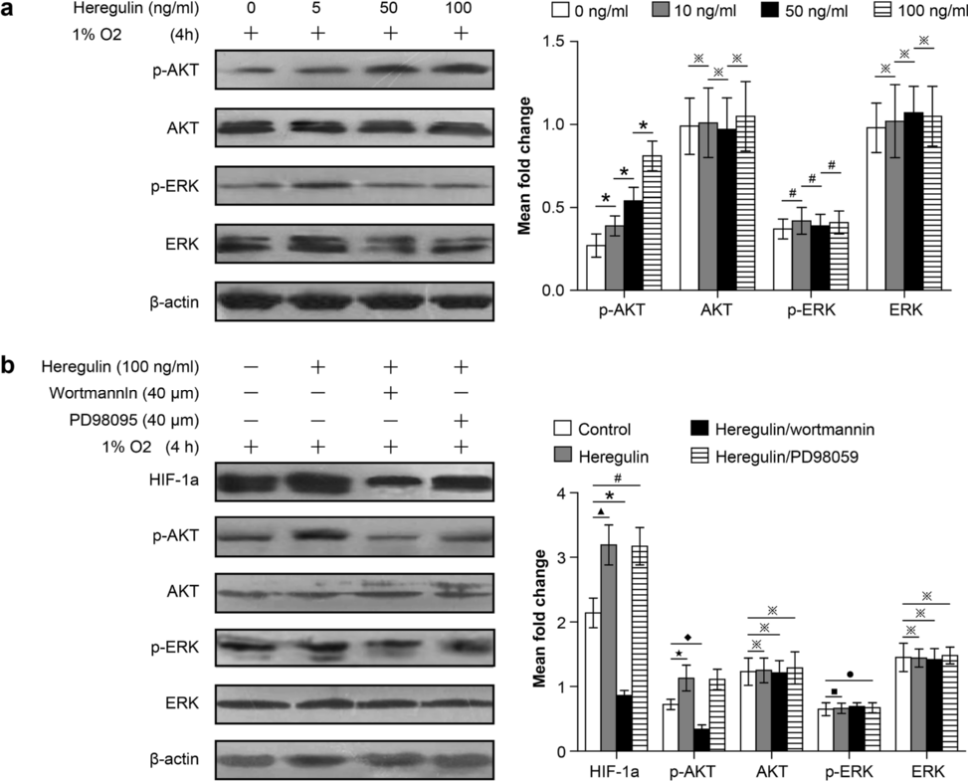
HIF-1a expression in SCLCs following heat treatment was regulated through the AKT but not the ERK signaling pathway. The method used was described in Fig. 6; NCI-H446-b subline cells are harvested and western blot analysis for protein expression is applied. **a** There were no obvious changes in AKT and ERK total protein expression levels (^※^ *p* > 0.05 between different groups). p-AKT was gradually induced with increasing heregulin concentrations (^*^ *p* < 0.05 between different groups), but p-ERK expression showed no significant change (^#^ *p* > 0.05 between different groups). **b** HIF-1a expression was upregulated after treated by heregulin (^▲^ *p* < 0.05 heregulin group vs control group), but was inhibited after co-treatment with wortmannin, but not PD98059 (^*^ *p* < 0.05 heregulin group vs heregulin + wortmannin group, ^**#**^ *p* > 0.05 heregulin group vs heregulin + PD98059 group). Total AKT and ERK protein levels also showed no significant changes between all treatment groups (^**※**^ *p* > 0.05 heregulin group vs heregulin + wortmannin group or heregulin + PD98059 group). p-AKT expression was upregulated when treated by heregulin (^★^ *p* < 0.05 heregulin group vs control group) and inhibited after co-treatment with wortmannin (^◆^ *p* < 0.05 heregulin group vs heregulin + wortmannin group). However, p-ERK expression in SCLCs was unaffected, regardless of heregulin or PD98059 treatment (^■^ *p* > 0.05 heregulin group vs control group, ^●^ *p* > 0.05 heregulin group vs heregulin + PD98059 group)

Because we found that the AKT and ERK signaling pathways were both involved in regulating HIF-1a expression in NSCLCs following hyperthermic treatment, we investigated the role of these pathways in HIF-1a regulation in SCLCs. As with NSCLCs, heregulin upregulated p-AKT expression in a dose-dependent manner, but had little effect on p-ERK expression (Fig. 7a). We pre-treated the NCI-H446-b cell subline with heregulin and then followed with wortmannin or PD98095, incubating under hypoxic conditions for 6 h. Our study showed that wortmannin also abolished heregulin induction of HIF-1a expression in the NCI-H446-b cell subline (Fig. 7b). Although PD98059 inhibits ERK, it had no significant effect on HIF-1a expression in SCLCs (Fig. 7b). These indicate that: (1) AKT signaling pathways help to regulate HIF-1a expression in both NSCLCs and SCLCs following hyperthermic treatment, and (2) the change in p-ERK expression has no significant relationship with HIF-1a expression in SCLCs following hyperthermia. Therefore, HIF-1a expression in SCLCs following hyperthermia is not regulated through the ERK signaling pathway.

## Discussion

Lung cancer is a heterogeneous, complex, and challenging disease to treat. Hyperthermia is an approach that takes advantage of the biological effects of heat to target tumors. Hyperthermia techniques have been the subject of extensive research with the overall goal of medicine development and equipment advancement [17]. Now, hyperthermia is regarded as an effective treatment for lung cancer, which may be as successful as surgery, radiotherapy, and chemotherapy. At present, clinical hyperthermia treatment methods for lung cancers include laser ablation, radiofrequency ablation, and magnetic hyperthermia [18]. Among these, radiofrequency ablation is becoming an increasingly accepted treatment for primary lung cancers in patients who are not candidates for subsegmental resection or lobectomy [19]. The prognosis for tumor patients has been closely correlated with activation and expression level of HIF-1a which are regulated by many factors such as DEC2 (differentiated embryonic chondrocyte gene 2) [20]. Besides this, heat treatment can cause hypoxia in the local tissue and increase HIF-1a expression levels, which can induce the over-proliferation of any residual tumors, leading to the recurrence of lung cancer [21]. Additionally, the angiogenesis potential increased by heat-induced hypoxia can also play a role in the rapid growth of residual tumor cells that have escaped heat ablation. This phenomenon is often referred to as a “malignant transformation” [22]. Hypoxia conditions present with, or induced by, heat treatment can be replicated in in vitro cell culture. The cell lines we used were already established and used in previous studies, so that further spontaneous transformation under normal culture conditions would be unlikely. Therefore, we concluded that the biological microenvironment associated with tumor growth changed due to the exposure to heat stress. Heat-adapted sublines became more proliferative, which became the focus of our subsequent work to identify the molecular/biological mechanisms involved in tumor recurrence.

In our previous study, HIF-1a was found overexpressed in many human cancers and various cell types, and the levels of HIF-1a activity were correlated with tumorigenicity, angiogenesis, and metastasis [23]. Its target gene, VEGF-A, is mainly regulated by HIF-1a at the transcriptional level, and VEGF-A also plays a critical role in tumor angiogenesis, growth, and metastasis. Targeting the HIF-1a/VEGF-A axis may be a promising strategy for combating tumor recurrences following hyperthermia treatment [24]. There has been a significant increase in the understanding of the importance of the signaling pathways that regulate the biological behavior of both NSCLCs and SCLCs (e.g. proliferation and angiogenesis) in recent years [25, 26]. Inhibitors of HIF-1a represent a new tool for improved cancer therapies [27]. A number of chemical, protein, and nucleic acid inhibitors are included and classified based on their mechanisms of inhibitory action. Among these, inhibiting upsteam signaling to block HIF-1a expression has proven effective [28]. HIF-1α is situated at the convergence of multiple oncogenic and tumor suppressor pathways, including the PI3K/AKT and MAPK/ERK pathways [29]. These signaling pathways are at the heart of a molecular signaling network that governs growth, proliferation, differentiation, and survival in many cell types [30]. They are dysregulated in various diseases, ranging from cancer to immunological, inflammatory, and degenerative syndromes, and thus represent an important drug target [31, 32]. A previous study found that activating the AKT and ERK signaling pathways could enhance HIF-1a/VEGF expression in some malignant tumors [33]. From the study about the mechanical regulation of signaling pathway in lung cancer, some scholars find that inactivation of Akt signaling can inhibit the growth of human lung cancer cells through reducing SP1 and p65 protein expression [34]. ERK and PI3K/AKT pathways can promote tumor cell growth and metastasis and a notable decrease of ERK and PI3K/AKT activation can be found in TRIM11 knocked down lung cancer cells [35]. These results are in congruence with our results that show inhibiting AKT and ERK expression through specific inhibitors in NSCLs can down-regulate HIF-1a expression and inhibit angiogenesis. However, inhibition of ERK expression had no significant effect on HIF-1a expression in SCLCs, which was regulated by the AKT signaling pathways. We hypothesized that the prime cause for this observation was that the regulatory mechanisms governing tumor properties are different, based on histological origin. Therefore, we intend to investigate these regulatory mechanisms further in the future.

Hyperthermia is a promising treatment for human lung cancer, but local recurrence is common, as growth of residual tumors can be induced by thermal ablation. Our study demonstrates that hyperthermia inherently changes the properties of cancer cells, facilitating the creation of different cancer sublines. These sublines exhibited enhanced viability and angiogenesis potential, and we determined that HIF-1a/VEGF-A plays a central role during this biological process. Furthermore, we investigated the molecular pathways induced/affected by hyperthermia. We found that HIF-1a expression was induced in heat adapted cell lines and that in NSCLCs this involves both PI3K/AKT and ERK signaling pathways, while only the PI3K/AKT signaling pathway is affected in SCLCs. Altogether, we hope that through investigating the molecular mechanisms of lung cancer recurrence following hyperthermia, our work will supply additional evidence for the use of multidisciplinary synthetic therapies to treat lung cancer.

## Conclusions

In this research we find that HIF-1a plays a critical role in the recurrence of lung cancer following hyperthermia treatment as the proliferation and angiogenesis potential of residual NSCLCs and SCLCs are induced by HIF-1a. However, HIF-1a expression in NSCLCs is regulated by both the AKT and ERK signaling pathway, but HIF-1a expression in SCLCs is regulated only by the AKT signaling pathway. Our study sheds light on the molecular regulatory mechanisms of lung cancer recurrence following hyperthermia treatment.

## Abbreviations

AKT, proteinkinase B; ERK, extracellular signal-regulated kinase; HIF-1a, hypoxia-inducible factor-1 alpha; NSCLC, non small cell lung cancer; SCLC, small cell lung cancer
